# Mechanism of action for N-substituted benzamide-induced apoptosis

**DOI:** 10.1038/sj.bjc.6600136

**Published:** 2002-03-18

**Authors:** A R Olsson, H Lindgren, R W Pero, T Leanderson

**Affiliations:** Section for Immunology, Department of Cell and Molecular Biology, BMC I:13, S-221 84, Lund, Sweden; Section for Tumor Immunology, Department of Cell and Molecular Biology, BMC I:12, S-221 84, Lund, Sweden

**Keywords:** apoptosis, caspase activity, Bcl-2, metoclopramide, declopramide

## Abstract

We have analysed the mechanism of action for induction of apoptosis by N-substituted benzamides using declopramide as a lead compound. We show here that declopramide at doses above 250 μM in the mouse 70Z/3 pre-B cell line or in the human promyeolocytic cancer cell line HL60 induced cytochrome *c* release into the cytosol and caspase-9 activation. The broad spectrum caspase inhibitor zVADfmk and caspase-9 inhibitor zLEDHfmk inhibited apoptosis and improved cell viability when administrated to cells 1 h before exposure to declopramide, whereas the caspase-8 inhibitor zIEDHfmk had less effect. Also, the over expression of Bcl-2 by transfection in 70Z/3 cells inhibited declopramide-induced apoptosis. Prior to the induction of apoptosis, a G_2_/M cell cycle block was induced by declopramide. The cell cycle block was also observed in the presence of broad spectrum caspase inhibitor zVADfmk and in a transfectant expressing high levels of Bcl-2. Furthermore, while p53 was induced in 70Z/3 cells by declopramide, neither the apoptotic mechanism nor the G_2_/M cell cycle block were dependent on p53 activation since both effects were also seen in p53 deficient HL60 cells after addition of declopramide.

*British Journal of Cancer* (2002) **86**, 971–978. DOI: 10.1038/sj/bjc/6600136 www.bjcancer.com

© 2002 Cancer Research UK

## 

Generally, cell and tissue homeostasis are regulated by genes that activate or suppress apoptosis, cell proliferation and differentiation (Raff, 1992). During the last decade a bulk of evidence has been assembled showing that apoptosis plays an essential roll in development, immune function, autoimmunity and cancer (Hanahan and Weinberg, 2000; Krammer, 2000; Meier *et al*, 2000). Likewise, several reports identify apoptotic cell death as an important cellular mechanism to be utilised in the treatment of tumours (Fisher, 1994; Reed, 1995; Houghton, 1999). Apoptosis is initiated by diverse death stimuli activating distinct biochemical pathways that converge into a common effector step, leading to cellular degradation (Cohen, 1997). The activation step can involve ligand interactions such as binding of Fas-ligand or TNF-α (tumour necrosis factor-α) to specific ectoplasmic death receptors belonging to the TNFR super family (Ashkenazi and Dixit, 1998) or oxidative stress and DNA damage induced by agents such as cisplatin or γ-radiation (Lowe *et al*, 1993; Henkels and Turchi, 1999). As a result of the apoptotic stimulus, the caspase cascade should be activated (Newton *et al*, 1998; Rich *et al*, 2000).

Caspases are a highly conserved cysteine protease family with specificity for aspartic acid residues in their substrates (Thornberry *et al*, 1997). The activation of the caspase cascade involves both the triggering as well as the final stages of apoptosis (i.e. the execution phase), and results in enzymatic cleavage of certain key substrates eventually leading to apoptotic-/programmed cell death (Thornberry, 1999; Hengartner, 2000). Two major triggering pathways have been defined for the caspase cascade. Mitochondria-mediated apoptosis involves the release of cytochrome *c* which binds to the Apaf-1 protein in the cytosol. This complex associates with procaspase-9 and forms the apoptosome (Beere *et al*, 2000), which in turn results in the activation of caspase-9 (Slee *et al*, 1999) and a subsequent activation of effector caspases -3, -6 and -7 (Hakem *et al*, 1998; Kuida *et al*, 1998; Thornberry, 1999). The Bcl-2 protein family are important regulators of caspase-9 activation, where the Bcl-2/Bax ratio in the mitochondria modulate the level of cytochrome *c* release after apoptotic stimuli (Adams and Cory, 1998; Hengartner, 2000). The other major apoptotic pathway important in immune cell homeostasis and autoimmunity, involve activation of caspase-8 via the death receptors localized on the cell surface and their interaction with FAAD (Fas associated death domain protein) (Juin and Evan, 2000). Once activated, caspase-8 can directly activate the effector caspases -3, -6 and -7 (Ashkenazi and Dixit, 1998). Also, as in the case of B cell receptor induced apoptosis, caspase-8 can induce apoptosis by processing the Bid protein into its truncated form that in turn can cause cytochrome *c* release from the mitochondria and consequently caspase-9 activation (Berard *et al*, 1999).

The N-substituted benzamide metoclopramide (MCA, neu-sensamide) and the structural analogue 3-chloroprocainamide (3CPA, declopramide) have recently been identified as compounds able to induce apoptosis *in vitro* (Pero *et al*, 1998; Liberg *et al*, 1999), and to have anti-inflammatory effects by inhibiting NFκB (nuclear factor kappa B) activity (Liberg *et al*, 1999) and TNFα production (Pero *et al*, 1999). Moreover, MCA and 3CPA have earlier been shown to have radio- and chemo-sensitising properties *in vivo* in murine and human tumour models (Kjellen *et al*, 1989, 1995; Olsson *et al*, 1997). 3CPA in combination with 5-FU (5-flourouracil) or cisplatin has recently completed phase-1 studies in patients with advanced stage cancers. At dose escalations up to total daily doses of 800 mg kg^−1^ alone or in combination with 5-FU, declopramide was considered safe and indicated efficacy. Due to the potential clinical value of declopramide as a chemo-sensitizer or as an anti-inflammatory agent, we further investigated the mechanism of action of N-substituted benzamides. We were able to demonstrate that these compounds induced a p53 independent G_2_/M cell cycle block and apoptotic cell death involving the mitochondrial pathway.

## MATERIALS AND METHODS

### Chemicals

Metoclopramide (MCA; neu-sensamide, metoclopramide hydrochloride monohydrate) and 3-chloroprocainamide (3CPA; declopramide, 3-chloroprocainamide hydrochloride) were custom synthesized by Oxigene Europe AB (Lund, Sweden) and prepared as hydrochloride salt solutions in phosphate buffered NaCl (0.9%).

Etoposide, cytochrome *c*, RNaseA, proteinase K, polybrene, PI (propidium iodide), 7AAD (7-amino-actinomycin D), and Nonidet P-40 were all purchased from Sigma-Aldrich Company (St. Louis, MO, USA). Z-VAD-fmk (benzyloxycarbonyl-Val-Ala-Asp-CH_2_F) was from Promega (Madison, WI, USA), Z-IETD-fmk (benzyloxycarbonyl-Ile-Glu(OMe)-Thr-Asp(OMe)-CH_2_F) and Z-LEHD-fmk (benzyloxycarbonyl-Leu-Glu(OMe)-His-Asp(OMe)-CH_2_F) were purchased from Calbiochem-NovaBiochem Corp. (Bad Soden, Germany), and Annexin V from Molecular Probes (Leiden, The Netherlands).

### Cells, transfection, and cell culturing

Human promyelocytic leukemia HL60 cells were maintained at 37°C, 5% CO_2_ and 80% humidity. The cell density was between 2 and 9×10^5^ cells per ml in RPMI 1640 medium supplemented with 10% foetal calf serum, gentamycin (50 μg ml^−1^), glutamine (2 mM), Na-pyruvate (2 mM), and HEPES-buffer (20 mM) (R-10 medium). The murine pre-B-cell line 70Z/3 was maintained under the same growth conditions, but the R-10 medium was complemented with 50 μM mercapto-ethanol. 70Z/3 cells were stably transfected with cDNA for mouse Bcl-2 (70Z/3^Bcl-2+^), inserted into a puromycin resistant mouse retro-virus vector (pBabe Puro Bcl-2 plasmid). The transfection was performed by incubation of cells with virus-containing supernatant in the presence of polybrene (4 μg ml^−1^, Sigma). Further cloning was performed with limiting dilution under selection by 0.75 μg ml^−1^ puromycin.

For apoptosis and flow cytometry experiments the cell density was adjusted to 2 to 5×10^5^ cells per well (HL60) or 2×10^5^ cells per well (70Z/3) at the day before the experiment.

### Flow cytometry

Apoptosis was measured by flow cytometry (FACScan®, Becton Dickinson, USA), and apoptotic cells were identified as cells that excluded the vital dye 7-amino-actinomycin D (7AAD^−^) or propidium iodide (PI^−^; Trevigen) but were positive to Annexin V (Alexa Flour 488) (Liberg *et al*, 1999). Annexin V recognize phosphatidyl serine moieties on the cell surface of apoptotic cells (Koopman *et al*, 1994). Drug-induced effects on the cell cycle was determined on cells prepared for flow cytometry analysis by labelling cells with propidium iodide according to Kallberg and Leanderson (1995). Flow cytometry analysis was performed after propidium iodide staining by incubation of cells for 30 min with Vindelövs low salt solution (3.4 mM Tris/HCl pH 7.6, 75 μM propidium iodide, 0.1% NP40, 10 mM NaCl) prior to analysis. The analysis was performed with a FACScan® analyzer (Becton Dickinson), where DNA content and grade of DNA fragmentation (apoptosis appearing as a sub-G_1_ fraction), and distribution of cells in G_1_, G_2_/M or S-phase, was determined.

### SDS–PAGE/Western blot analysis

SDS–PAGE and Western blot analysis was performed on the 10 000 **g** supernatant fraction from total soluble proteins (Wang *et al*, 1998), or on cytosolic and mitochondrial fractions prepared from HL60 and 70Z/3 cells. Mitochondria enriched fractions were prepared by 60 s of gentle homogenisation with a plastic Eppendorf pestle in an Eppendorf tube (100 μl cell suspension representing 5×10^6^ cells suspended in homogenising buffer; HEPES-KOH 20 mM, pH 7.5, 10 mM KCl, EDTA 1 mM, EGTA 1 mM, DTT 1 mM, PMSF 0.1 mM and sucrose 250 mM) (Yang *et al*, 1997) before centrifugation at 700 **g**. This was followed by a second centrifugation at 10 000 **g** to separate the mitochondria from the cytosol fraction. Electrophoresis was performed on a 12% polyacrylamide gel (150 V per 60 mA). Equal amounts of protein (30–60 μg) were loaded in each lane. Protein transfer to a nylon membrane (Hybond-C Extra; Amersham, UK) was performed at 15 V per 200 mA for 30 min. After blocking overnight in 5% dry fat-free milk in TBST (10 mM Tris-HCl, pH 8, 150 mM NaCl, 0.05% Tween-20), incubation with a primary antibody for 2 h was performed. Cytochrome *c* was detected using a cytochrome *c* antibody (mouse IgG2b, clone 7H8.2C12; Pharmingen). To detect pro-caspase-9 or active caspase-9, a rabbit polyclonal anti-mouse caspase-9 (Santa Cruz) or a rabbit polyclonal anti-human caspase-9 (Pharmingen) was used. The Bcl-2 antibody was purchased from Pharmingen, anti-actin from Sigma, the rabbit polyclonal anti-mouse-p53 antibody (NCL-p53-CM5p) was purchased from Novocastra Laboratories (UK) and the anti-human/-mouse p53 (mouse monoclonal IgG1, Pab 240) was from Santa Cruz. After washing in TBST incubation with either a secondary (HRP)-conjugated anti-mouse, -rabbit or -goat antibody (Santa Cruz) was performed, followed by chemiluminescence ECL-reagent (Amersham).

## RESULTS

### 3CPA-induced apoptosis correlates with cytochrome *c* release and caspase-9 activation

We have reported earlier that the N-substituted benzamides metoclopramide (MCA) and declopramide (3CPA) have the ability to induce apoptosis in target cells such as murine 70Z/3 and human HL60 cells *in vitro* (Pero *et al*, 1998; Liberg *et al*, 1999). To further define the cytotoxic mechanism of N-substituted benzamides, we expanded our previous studies using the murine 70Z/3 pre-B cell line as the *in vitro* model (Liberg *et al*, 1999) and 3CPA as the active moiety. The common apoptotic pathway leading to drug-induced apoptosis is preceded by a disruption of the mitochondrial trans-membrane potential, followed by cytochrome *c* release into the cytosol and activation of caspase-9 (Houghton, 1999; Kroemer and Reed, 2000).

**Figure 1:**
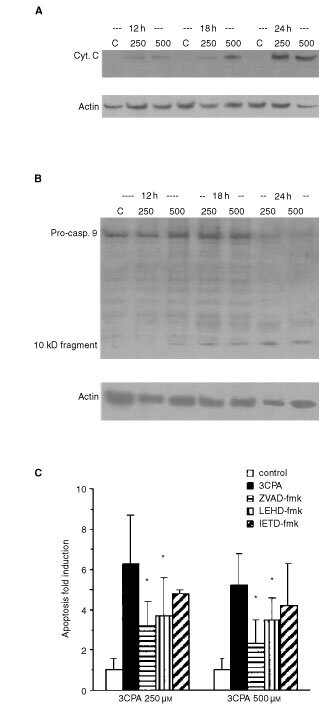
Induction of apoptosis in 70Z/3 cells treated with 3CPA at 250 μM and 500 μM. (**A**) Cytochrome *c* release into the cytosol after exposure to 3CPA for indicated time periods measured by Western blot analysis. (**B**) Pro-caspase-9 processing determined in cytosolic fractions after exposure to 3CPA. Actin was used as an internal standard of the protein load and the results shown are representatives of two separate experiments. (**C**) Caspase inhibitors reduce 3CPA-induced apoptosis in 70Z/3 cells. Apoptosis, measured as AnnexinV^+^7AAD^−^ cells, was determined after exposure to 3CPA at 250 μM (left) and 500 μM (right) for 24 h. The pan-caspase inhibitor ZVAD-fmk (50 μM, *n*=4), the caspase-9 inhibitor LEHD-fmk (50 μM, *n*=3), or the caspase-8 inhibitor IETD-fmk (50 μM, *n*=3) were added 1 h before treatment with 3CPA. The level of apoptosis is expressed relative to the level in cell cultures treated with the inhibitor only. Results show mean±s.d., and (*) indicates a statistical difference compared to 3CPA only (paired *t*-test, *P*<0.05).

For that reason, we performed Western blot analysis of cytosol preparations from 70Z/3 cells exposed to 3CPA. Cytochrome *c* release into the cytosol could be detected after 12 h incubation with 3CPA (Figure 1A

). The lack of detectable cytochrome *c* in cytosol fractions from unexposed cell cultures, was taken as evidence that no leakage from the mitochondria due to bad preparation procedure occurred (Figure 1A). Processing of procaspase-9 into active caspase-9, as detected by the disappearance of the 48 kD procaspase-9 band in conjunction with the appearance of a band at 10 kD, was seen after 12–18 h (Figure 1B). Moreover, the addition of 3CPA (250 and 500 μM) to 70Z/3 cells, induced an increase of apoptotic cells above background as detected by Annexin V^+^7AAD^−^ staining after 6–12 h. The fraction of early apoptotic cells reached a steady state level of 10–15% Annexin V^+^7AAD^−^ cells after 18–24 h (data not shown). As shown in Figure 1C, co-administration for 24 h of the pan-caspase inhibitor Z-VAD-fmk (50 μM) significantly reduced the fraction of Annexin V^+^7AAD^−^ cells (*P*<0.05, paired *t*-test, *n*=4). The caspase-9 inhibitor Z-LEHD-fmk (50 μM) also significantly inhibited 3CPA-induced apoptosis (*P*<0.05, paired *t*-test, *n*=3), whereas addition of the caspase-8 inhibitor Z-IETD-fmk (50 μM) (Thornberry *et al*, 1997; Garcia-Calvo *et al*, 1998) had less effect (*P*>0.1, paired *t*-test, *n*=3) (Figure 1C). We concluded from these experiments that the cytotoxic function of N-substituted benzamides required a functional caspase-9 activation pathway, although the involvement of caspase-8 activity in 3CPA-induced apoptosis could not be totally excluded.

### Cytochrome *c* release and caspase-9 activation are inhibited by the over-expression of Bcl-2

**Figure 2:**
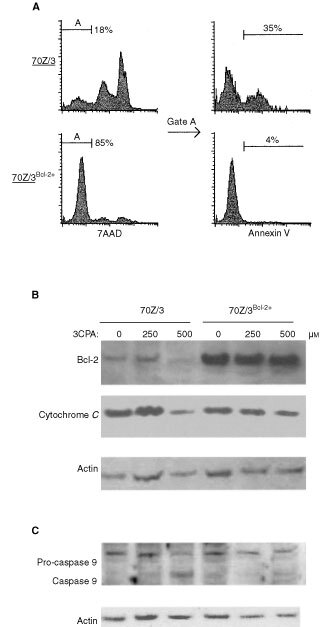
Over-expression of Bcl-2 in 70Z/3 cells inhibits 3CPA-induced apoptosis. (**A**) FACS-analysis of 70Z/3 (upper panel) and 70Z/3^Bcl-2+^ (lower panel) cells after exposure to 3CPA (500 μM) for 18 h. 7AAD negative (viable) cells were gated (gate A) and analysed for Annexin V^+^ expression (% of gated cells). (**B**) Levels of Bcl-2 and cytochrome *c* expression were determined by Western blot analysis in mitochondrial enriched fractions after 18 h incubation with 3CPA at the indicated concentrations (30 μg protein per lane). (**C**) Pro-caspase-9 processing into active caspase-9 was determined in the cytosolic fraction after 18 h incubation with 3CPA at the indicated concentrations. Actin was used as an internal standard to control the amount of cytosolic protein loaded in each lane. Results shown are representatives of two independent experiments.

Cytochrome *c* release and caspase-9 activation are to some extent regulated by members of the Bcl-2 protein family, which are associated with the exterior mitochondria membrane. The mitochondrial trans-membrane potential, and thereby membrane integrity, is at least in part regulated by the ratio between the anti-apoptotic Bcl-2 family proteins Bcl-2 and Bcl-X_L_ and the pro-apoptotic proteins Bax and Bad (Oltvai *et al*, 1993). Thus, to study the influence of Bcl-2 on 3CPA-induced apoptosis, 70Z/3 cells were transfected with a Bcl-2 expression vector and a stable 70Z/3^Bcl-2+^ cell line was established. As shown in Figure 2A

, treatment with 3CPA at a dose of 500 μM did not induce apoptosis or cyto-toxicity to the same extent in 70Z/3^Bcl-2+^ cells as in wild-type 70Z/3 cells. Furthermore, Western blot analysis showed that Bcl-2 levels in the mitochondria-enriched fraction remained on an equally high level independently of dose in 70Z/3^Bcl-2+^ cells. In contrast, the Bcl-2 levels tended to decline in the mitochondria-enriched fraction of wild-type 70Z/3 cells treated with 500 μM 3CPA for 18 h (Figure 2B). Also, release of cytochrome *c* from mitochondria and caspase-9 activation was seen after 18 h 3CPA treatment in 70Z/3 cells but was not observed after treatment of 70Z/3^Bcl–2+^ cells (Figure 2B,C). These experiments strongly suggested that N-substituted benzamides induced an apoptotic response via a mechanism involving the mitochondrial pathway.

### 3CPA treatment induce a G_2_/M cell cycle block in 70Z/3 cells

**Figure 3:**
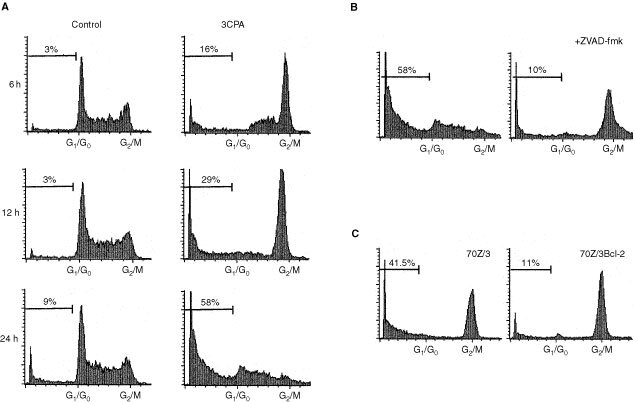
3CPA induces cell cycle block in the G_2_/M-phase in 70Z/3 cells independent of caspase activity. (**A**) 70Z/3 cells were exposed for 3CPA at 500 μM for 6 to 18 h whereafter cells in different cell cycle phases were determined by flow cytometry analysis of propidium iodide labelled cells (Kallberg and Leanderson, 1995). Indicated numbers in the figure show the sub-G_1_ fraction expressed as percent of total events. (**B**) ZVAD-fmk (50 μM) prevents 70Z/3 cells to undergo apoptosis after 24 h of exposure to 500 μM 3CPA and allow cells to accumulate in G_2_/M-phase. Addition of ZVAD-fmk only had no effect on cell cycle distribution (data not shown). (**C**) Cell cycle arrest in G_2_/M phase occurs regardless of the inhibition of apoptosis in 70Z/3^Bcl-2+^ cells after 12 h exposure to 500 μM 3CPA. One out of two representative experiments is shown.

Drug-induced apoptosis via cytochrome *c* release and caspase-9 activation often involve upstream events associated to oxidative stress and DNA damage (Beere *et al*, 2000; Rich *et al*, 2000). Moreover, cell cycle regulatory events resulting in cell cycle arrest and mitochondria-associated apoptosis can be mediated by a number of cellular signalling pathways. In order to investigate if 3CPA affected the cell cycle we analysed the cell cycle distribution of 70Z/3 cells after 3CPA treatment. As shown in Figure 3A

, an increased fraction of cells in G_2_/M-phase was seen already after 6 h incubation with 500 μM 3CPA, followed by apoptosis and the formation of a sub-G_1_ fraction after 12–24 h. Thus, an accumulation of cells in G_2_/M-phase occurred prior to apoptosis as detected by the accumulation of a sub-G_1_ population.

Next we wanted to establish that the increased fraction of cells in G_2_/M-phase after exposure to 3CPA was a true cell cycle block and not just due to the fact that cells in this phase were protected from cell death whereas cells in G_0_/G_1_/S-phase were not. Furthermore, we wanted to investigate whether caspase activation was needed for the establishment of the G_2_/M-phase blockade. To this end, we added the caspase inhibitor ZVAD-fmk (50 μM) together with 3CPA (500 μM) to 70Z/3 cells and performed cell cycle analysis after 24 h of incubation (see Figure 3A for kinetics of the G_2_/M blockade). As shown in Figure 3B, over 50% of the events in the control cultures were in the sub-G_1_ fraction and the remaining population more or less evenly spread throughout the cell cycle. However, in ZVAD-fmk treated cultures only 10% of the events were in the sub-G_1_-region and the G_2_/M block was still easily observed after 24 h of incubation. Furthermore, 3CPA treatment of 70Z/3^Bcl-2+^ cells also induced a G_2_/M block even though induction of apoptosis in this cell line was marginal after 12 h showing that Bcl-2 does not affect the G_2_/M block (Figure 3C). Thus, we concluded that 3CPA treatment induced a cell cycle block in the G_2_/M-phase of the cell cycle, and the establishment of this cell cycle block was independent of ZVAD-fmk responsive caspase activation. On the other hand, the G_2_/M block did not cause apoptosis by itself but seemed to need a functional mitochondria-mediated caspase pathway down-stream. Thus, if the cell cycle block is a trigger of caspase activation or if the two are independent events remains to be established.

### The induction of apoptosis by N-substituted benzamides is not dependent of p53

**Figure 4:**
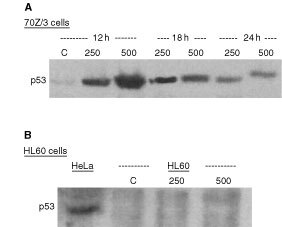
3CPA induces p53 in 70Z/3 cells but not in HL60 cells. (**A**) p53 expression in mouse 70Z/3 cells after incubation with 3CPA at 250 μM or 500 μM. p53 expression in the cytosol was detected by the rabbit polyclonal anti-mouse-p53 antibody (NCL-p53-CM5p; Novocastra Laboratories) (40 μg protein/lane). (**B**) Lack of p53 protein expression in HL60 cells exposed to 250 or 500 μM 3CPA for 12 h. p53 in the cytosol was detected by the mouse monoclonal anti-human/-mouse p53 antibody (Santa Cruz, sc-240). Human HeLa cells exposed to 500 μM 3CPA were included as a positive control and equal amount of protein (47 μg) was loaded.

A possible adjacent mediator between cell cycle arrest and apoptosis is the nuclear p53 protein. p53 is up-regulated by DNA damage and has been shown to be associated with cell cycle block both in G_0_/G_1_- and G_2_/M-phase depending on the nature of damage, dose and cell type (Lu and Lane, 1993). We therefore investigated the possible effect of 3CPA on p53-expression by Western blot analysis of cell extracts from 70Z/3 cells exposed to 250 or 500 μM 3CPA for 12–24 h. As shown in Figure 4A

, a substantial up-regulation of p53 was detected in the mitochondria-enriched fraction at 12 h, and the elevated p53 levels declined with time to slightly above the control level after 24 h. The data suggested that 3CPA-induced G_2_/M blockade might involve the p53-signalling pathway. For that reason, we decided to expand our studies to the p53 deficient HL60 cell line in which we could directly analyse whether both the ability of N-substituted benzamides to induce apoptosis and G_2_/M arrest were dependent on p53 signalling.

**Figure 5:**
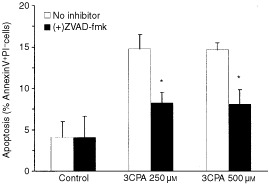
3CPA-induced apoptosis in p53-negative HL60 cells. Apoptosis was determined as percent AnnexinV^+^PI^-^ cells of total cells in culture, after exposure to 3CPA at 250 and 500 μM for 18 h. The level of apoptosis was reduced by pre-incubation with the pan-caspase inhibitor ZVAD-fmk (50 μM) for 1 h before addition of 3CPA. Bar-graphs represent the average±s.d. of three independent experiments, and (*) indicates a statistical difference compared to 3CPA only (paired *t*-test, *P*<0.05).

**Figure 6:**
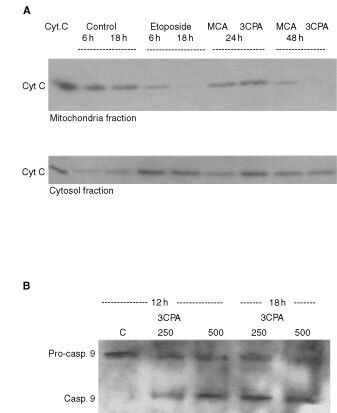
N-substituted benzamides induce cytochrome *c* release and activation of caspase-9 in HL60 cells. (**A**) Cytochrome *c* release induced by MCA (500 μM) and 3CPA (250 μM) in HL60 cells after 24 and 48 h, measured by Western blot analysis of mitochondria or cytosol fractions (30 μg protein/lane). Cells were incubated with etoposide for 6 and 18 h at 100 μM as a positive control. (**B**) Activation of caspase-9 after treatment of HL60 cells with 3CPA at 250 and 500 μM for 12 and 18 h (48 μg protein per lane). One representative experiment out of two is shown.

As expected, we were not able to detect p53 expression in human leukaemic HL60 cells after treatment with 3CPA, since HL60 cells are known to lack a functional p53 pathway (Danova *et al*, 1990) (Figure 4B). However, when HL60 cells were exposed to 3CPA, an apoptotic cell fraction was observed by FACS analysis (Annexin V^+^PI^−^ cells), showing that the ability of this compound to induce apoptosis was not requiring a functional p53 in this cell line (Figure 5

). The apoptotic cells appeared in culture 12–18 h after the compounds were added, which was consistent with earlier data obtained in HL60 cells (Pero *et al*, 1998), and the induction of apoptosis was inhibited by the presence of ZVAD-fmk at 50 μM (Figure 5). Also, cytochrome *c* release from the mitochondria to the cytosol was detected after treatment of HL60 cells with 3CPA or MCA (Figure 6A

). In conjunction with cytochrome *c* release after 12 h (data not shown), procaspase-9 was processed into its active form caspase-9 after 12–18 h when exposed to 3CPA at doses of 250 and 500 μM at 24–48 h (Figure 6B). Thus, we conclude from these results that N-substituted benzamides can induce apoptosis via a p53-independent mechanism.

**Figure 7:**
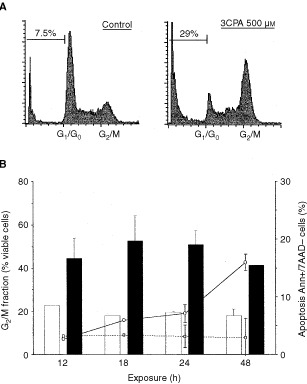
3CPA-induces apoptosis and cell cycle block in human HL60 cells. (**A**) Cell cycle distribution in HL60 cells exposed to 500 μM 3CPA for 24 h. Cells in different cell cycle phases were determined by flow cytometry analysis of propidium iodide labelled cells. Indicated numbers in the figure show the sub-G_1_ fraction expressed as percent of total events. (**B**) The accumulation of HL60 cells in G_2_/M-phase appears before apoptosis is induced. HL60 cells were incubated with 500 μM 3CPA for indicated time points before cell cycle analysis and apoptosis was measured. Bars represent G_2_/M fractions expressed as % of total cells±s.d. in, (□) unexposed cells and (▪) cells exposed to 3CPA for indicated time periods. Apoptotic cells were determined as Annexin^+^7AAD^-^ cells (% of total cells±s.d.) not exposed (–□–) or exposed (○) to 500 μM 3CPA for indicated time periods.

Lastly we analysed the effect of 3CPA addition on the cell cycle distribution of HL60 cells. As shown in Figure 7A

, a G_2_/M block was easily detectable in 3CPA-treated cultures. The accumulation of cells in G_2_/M-phase took place before cells expressed phosphatidyl serine on their cell surface (Annexin V^+^7AAD^−^ cells) and appeared as a sub-G_1_ population with less DNA than viable G_0_/G_1_-cells (Figure 7B). Hence, also the effect on cell cycle distribution after 3CPA treatment can be established in the absence of p53.

## DISCUSSION

The N-substituted benzamides have been shown to have multiple modes of biological activity, because they: (i) have D_2_- and 5-HT_3_-receptor binding affinity (Harrington *et al*, 1983); (ii) induce apoptosis and inhibit proliferation *in vitro* (Pero *et al*, 1998; Liberg *et al*, 1999); (iii) sensitise low dose radiation and delay *in vivo* tumour growth (Hua and Pero, 1997; Olsson *et al*, 1997); (iv) have anti-inflammatory properties by inhibiting NFκB activity in human Jurkat T-cells (Lindgren *et al*, 2001) and mouse pre-B 70Z/3 cells (Liberg *et al*, 1999) and finally; (v) inhibit TNFα production *in vitro* as well as *in vivo* (Pero *et al*, 1999). To eliminate species specific responses we have utilised two different haematopoetic cell lines, 70Z/3 mouse pre-B-cells and human HL60 promyelocytic leukaemia cells, to study the mechanism of apoptosis induced by N-substituted benzamides. These cell lines differ in origin and they have several distinct differences in gene expression and function. Anticancer drug-induced apoptosis has been shown to depend on intact caspase cascades (Li *et al*, 1997; Ferrari *et al*, 1998). In the current investigation the pan-caspase inhibitor ZVAD-fmk inhibited apoptosis induced by 3CPA both in 70Z/3 and HL60 cells (Figures 1C and 5). Furthermore, the specific caspase-9 inhibitor LEDH-fmk (Thornberry *et al*, 1997; Garcia-Calvo *et al*, 1998; Komoriya *et al*, 2000) inhibited apoptosis almost at the same level (30–50% inhibition), while the specific caspase-8 inhibitor IETD-fmk (Thornberry *et al*, 1997; Komoriya *et al*, 2000) showed less inhibitory effect on 3CPA-induced apoptosis (Figure 1C). The data suggest to us that caspase-9 is the initiator caspase involved in 3CPA-induced apoptosis. The conclusion is further supported by the observation that the addition of 3CPA induces cytochrome *c* release to the cytosol and caspase-9 activation. Hence, we believe that we have defined an apoptotic pathway involved in N-substituted benzamide-induced apoptosis.

Another interesting observation in this study is the distinct G_2_/M cell cycle block induced by 3CPA. By using caspase inhibitors and a Bcl-2 transfectant cell line we could demonstrate that this cell cycle arrest is an event occurring prior to 3CPA-induced apoptosis. It is well documented that DNA damage induced by γ-radiation (Clarke *et al*, 1993; Radford *et al*, 1994), oxidative stress (Beere *et al*, 2000), chemicals such as glucocorticoids (Meng and El-Deiry, 2001), cisplatin (O'Connor and Lu, 2000) or topoisomerase-2 inhibitors (Dubrez *et al*, 1995), cause cell cycle arrest in G_0_/G_1_ or G_2_/M-phase prior to cell death via apoptosis. Whether any of these routes to apoptotic cell death are activated by N-substituted benzamides remains to be established.

It has been shown that DNA damage induced by ionising radiation involves activation of p53, and can result in cell cycle arrest both in the G_1_/G_0_-phase and in G_2_/M-phase in 70Z/3 cells (Aloni-Grinstein *et al*, 1995). Indeed, in analogy with γ-radiation, we measured an up-regulation of p53 in 70Z/3 cells after treatment with 3CPA (Figure 4A). However, it is not likely that p53 plays an essential role in the apoptotic pathway activated by the N-substituted benzamides since apoptosis also was induced in the p53 deficient HL60 cell line. Furthermore, a G_2_/M cell cycle block was also observed in HL60 cells, arguing for the fact that the benzamide-induced cell cycle block is established by signalling pathways distinct from p53.

In conclusion, we have shown that N-substituted benzamides can activate the caspase cascade via the mitochondrial pathway and arrest cells in the G_2_/M phase. If the induction of apoptosis by the N-substituted benzamides is dependent on cell cycle arrest or not, is still not resolved and the connection between these two events must be subject to further studies.
